# Autonomous adaptive data acquisition for scanning hyperspectral imaging

**DOI:** 10.1038/s42003-020-01385-3

**Published:** 2020-11-18

**Authors:** Elizabeth A. Holman, Yuan-Sheng Fang, Liang Chen, Michael DeWeese, Hoi-Ying N. Holman, Paul W. Sternberg

**Affiliations:** 1 Division of Chemistry and Chemical Engineering, California Institute of Technology, Pasadena, CA USA; 2 Department of Physics, University of California, Berkeley, CA USA; 3 Division of Molecular Biophysics and Integrated Bioimaging, Lawrence Berkeley National Laboratory, Berkeley, CA USA; 4 Division of Biology and Biological Engineering, California Institute of Technology, Pasadena, CA USA

**Keywords:** Optical imaging, Data acquisition

## Abstract

Non-invasive and label-free spectral microscopy (spectromicroscopy) techniques can provide quantitative biochemical information complementary to genomic sequencing, transcriptomic profiling, and proteomic analyses. However, spectromicroscopy techniques generate high-dimensional data; acquisition of a single spectral image can range from tens of minutes to hours, depending on the desired spatial resolution and the image size. This substantially limits the timescales of observable transient biological processes. To address this challenge and move spectromicroscopy towards efficient real-time spatiochemical imaging, we developed a grid-less autonomous adaptive sampling method. Our method substantially decreases image acquisition time while increasing sampling density in regions of steeper physico-chemical gradients. When implemented with scanning Fourier Transform infrared spectromicroscopy experiments, this grid-less adaptive sampling approach outperformed standard uniform grid sampling in a two-component chemical model system and in a complex biological sample, *Caenorhabditis elegans*. We quantitatively and qualitatively assess the efficiency of data acquisition using performance metrics and multivariate infrared spectral analysis, respectively.

## Abstract (web summary)

Holman et al. develop a grid-less autonomous adaptive sampling method to explore high-dimensional spatiochemical experimental systems. Their method greatly decreases image acquisition time while improving spatial resolution, and when implemented with FTIR, it outperforms existing standard grid sampling approaches. They further show its utility for a complex biological sample, *C. elegans*.

## Introduction

Advancements in optical microscopy, especially fluorescence microscopy, have enabled biologists to observe multiplexed living cellular events with ever higher spatial and temporal resolutions^1^. The use of targeted fluorescent indicators provides spatial and temporal context to omics analyses^2–4^, resulting in discoveries of dynamic spatial architecture in disease pathogenesis^5^, organogenesis^6^, and wound healing^7^. These advances inspired the drive towards high-dimensional image-based profiling^8^, which requires high information-content, rapid, robust measurements of as many living cell or tissue phenotypes as possible to capture time-dependent spatial heterogeneities in structure and morphological patterning.

One solution is to introduce another complementary dimension of label-free observation one that focuses on the spatiochemical mapping of biological systems. This information can be used to guide fluorescence microscopy, its real-time imaging capabilities limited to a few features of interest identified a priori, and to improve the interpretation of omics data and information from advanced transmitted light microscopy images. Non-invasive and label-free multiplexed imaging techniques, such as scanning synchrotron radiation-based Fourier transform infrared (SR-FTIR) spectromicroscopy, can identify and monitor spatial heterogeneity in chemical composition that is indistinguishable using the visible region of the electromagnetic spectrum; however, a major challenge in using these techniques for real-time characterization of time-dependent biochemical processes is the substantial image acquisition times that ranges from minutes to hours. This complication emerges from the high dimensionality of the generated data set, which contains not only spatial but also spectral information, and the utilization of uniform grid (UG) sampling as the current standard sampling method, which historically is objective and computationally inexpensive.

With advancements in the accessibility of computing technology, we find that grid-less autonomous adaptive data acquisition (AADA) is a viable and more efficient alternative to UG sampling. AADA maintains a systematic and reproducible approach while capturing spectral and spatial heterogeneity with fewer sampled points and shorter experimental time frames. We discuss the significance of this method for studying time-sensitive living systems and its future development towards monitoring time-dependent phenomena in biological systems prior to expanding our discussion towards AADA’s applicability to other fields and workflow processes.

## Results

### Implementation of AADA

**Fig. 1:**
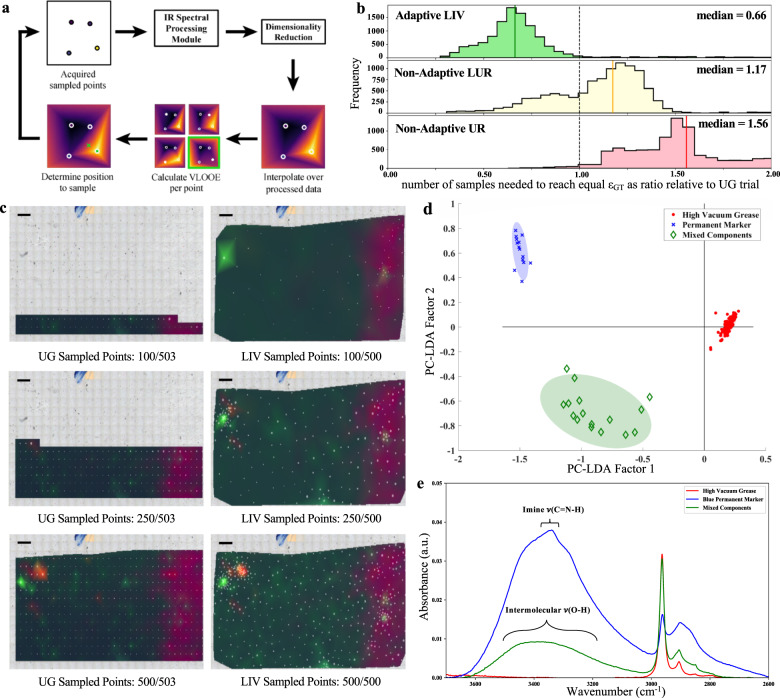
Implementation and performance evaluation of autonomous adaptive LIV data acquisition using a simple two-component abiotic model system. **a** Autonomous adaptive sampling workflow. **b** Eleven thousand simulated trials, performed 1000 times over each biologically independent *C. elegans* map (*n* = 11), were used to identify number of samples required to reach equal *ϵ*_GT_ for LIV, LUR, and UR methods are plotted in histograms as a ratio with respect to UG sampling. A ratio of 1 is equivalent performance (dotted line); 92.6% of LIV trials, 27.1% of LUR trials, and 1.6% of UR trials outperformed the relative UG trial in simulated sampling. **c** Autonomous sampling of abiotic two-component sample for current standard UG and adaptive LIV methods (scale bar, 200 μm). First three PCA components are shown as an RGB overlay with sampled points (white circles) and their predictive error (circle diameter). **d** Exploratory 2D PC-LDA score plot of noise-filtered and baseline-corrected acquired data 95% confidence intervals as shaded areas. **e** Globar FTIR spectral mean of each identified PC-LDA cluster showing the peak identifications used to corroborate chemical identity of high vacuum grease (red), permanent marker (blue), and mixed components (green).

To implement an autonomous adaptive sampling algorithm (Fig. 1a) for data acquisition, we prioritized optimization of spatiotemporal and spatiochemical sampling efficiency while operating under experimental parameters that nonetheless yield comprehensive and informative spectral map data. We assume that less predictable yet detectable phenomena emerging from spatiochemical heterogeneity are primary regions of interest, informational “hot spots” that should be spatiochemically resolved with subsequent sample points after initial detection. To achieve this, our adaptive sampling is driven by leave-one-out cross-validation (LOOCV)^9^ to facilitate rapid and accurate approximations of the experimentally mapped space^10^ for predictive error calculations from which the algorithm can rapidly identify regions for subsequent sample exploitation^11–13^. We build our surrogate models using a hybrid sequential sampling strategy closely related to other established methods^11,14,15^ by combining Two-dimensional (2D) barycentric linear interpolation with Voronoi tessellation (LIV). With LIV, the relative importance of a sampled point is determined by its Voronoi-weighted leave-one-out error (*ϵ*_LOO_)^12,16^, which is calculated by normalizing and equally weighing LOO with the Voronoi predictive error. Since collected IR spectra often form continuous and multimodal regions in our input space per sampled point^12^, we introduced an IR spectral preprocessing module upstream of our surrogate model construction and LOOCV to conserve the spectral resolution while the algorithm determines where to subsequently sample.

### Simulation-based evaluation of adaptive sampling performance

To assess adaptive LIV sampling algorithm performance prior to our experiments, we performed preliminary simulations using 11 previously collected, spatially high-resolution, broadband SR-FTIR spectral maps of different *Caenorhabitis elegans* (*C. elegans*) strains, our final experimental system. We assumed each complete map to be the “ground truth” upon which we compared four different sampling strategies: non-adaptive UG, uniform random (UR), least unexplored region (LUR), and adaptive LIV simulated subsampling methodologies. We calculated our performance metric of ground truth error (*ϵ*_GT_), a value that measures the error between sampling method’s interpolation and its corresponding complete high-resolution map, to enable quantitative evaluations and comparisons among the sampling strategies. When benchmarking the aforementioned methodologies against UG sampling (Fig. 1b), we find that although UG sampling does perform better than other non-adaptive sampling methods, it is significantly outperformed by our adaptive LIV sampling–LIV required 66% of the sampled points that UG needed to achieve equal *ϵ*_GT_.

### AADA for imaging a two-component abiotic system

As our first experimental demonstration, we designed a two-component chemical model system of blue permanent marker and high vacuum grease for spatiochemically resolved characterization using scanning FTIR spectroscopy. This complete sample characterization enabled quantitative evaluations and comparisons between adaptive LIV and widely utilized, non-adaptive UG data acquisition (Fig. 1c). In this visibly featureless case, the mapped domain was selected with minimal experimenter knowledge input to guide the autonomous adaptive data acquisition. Under these experimental conditions, we quantitatively and qualitatively determine data acquisition performance using mathematical and spectral metrics. When using mean Voronoi-weighted LOO ${\langle {\epsilon }_{\mathrm{LOO}}\rangle }_{V}$ to quantify modeling accuracy, we found that adaptive LIV data acquisition outperformed the non-adaptive data acquisition methods (Supplementary Fig. 1) in this experimental system. To verify this conclusion, we tuned the spectral, on-target ratio (OTR) assessment by selecting the major contributing peak per component using our normalized mean standard spectra (Supplementary Fig. 2) and variance spectra (Supplementary Fig. 3); peak selection guided by normalized spectra emphasize chemical identification^17^ over concentration in spectral interpretation. For high vacuum grease, we referenced the symmetric stretching mode of *ν*(Si-O-Si) at 798 cm^−1^ emerging from its fumed amorphous silica^18^ composition. For permanent ink presence, we used the major peak at 1580 cm^−1^ stemming from conjugated carbon–carbon ring *ν*(-C=C-) stretching modes^19^ in pigment compounds^20^, which was further substantiated by the presence of aromatic *ν*(=C-H) vibrations between 3105 and 3000 cm^−1^ ^19^ (Supplementary Fig. 4). All spectra were evaluated for non-adaptive UG and adaptive LIV experiments prior to processing the OTR as the proportion of on-target sampled points to total sampled points. Using this spectrally based metric for enhanced real-world fidelity^21^, we confirmed that adaptive LIV data acquisition (OTR = 0.95) outperforms non-adaptive UG (OTR = 0.19) data acquisition in experimental cases where domain knowledge is either limited or unavailable.

To verify that our acquired adaptive LIV data is interpretable through multivariate analysis from an experimenter’s standpoint, we performed principal component analysis (PCA) followed by linear discriminant analysis (LDA)^22^ on the noise-removed IR data to discriminate between the permanent marker and high vacuum grease present in the spatiochemical map (Fig. 1d). We see that the first PC-LDA factor distinguishes between permanent ink-containing spectral regions and pure high vacuum grease, while the second PC-LDA factor separates between pure permanent ink and regions containing both permanent ink and high vacuum grease. This conclusion is further supported by the mean spectra plotted per cluster (Fig. 1e); we see the imine *ν*(C=N-H) from 3400 to 3300 cm^−1^ and intermolecular hydrogen-bonded *ν*(O-H) at 3550 and 3230 cm^−1^ contributions^19^ from permanent ink’s pigment compounds and alcohols, respectively. The identified high vacuum grease cluster matches the standard mean spectra expectations with vibrational silence in frequencies >3000 cm^−1^, while peak broadening and the change in peak ratio between the imine and intermolecular hydrogen bonding regions of the permanent ink and mixed component clusters suggest that permanent ink alcohols experienced inhibited evaporation in the mixed component regions due to the ink’s deposition under the high vacuum grease during sample preparation (Supplementary Fig. 5).

### AADA for imaging living multicellular organisms

**Fig. 2:**
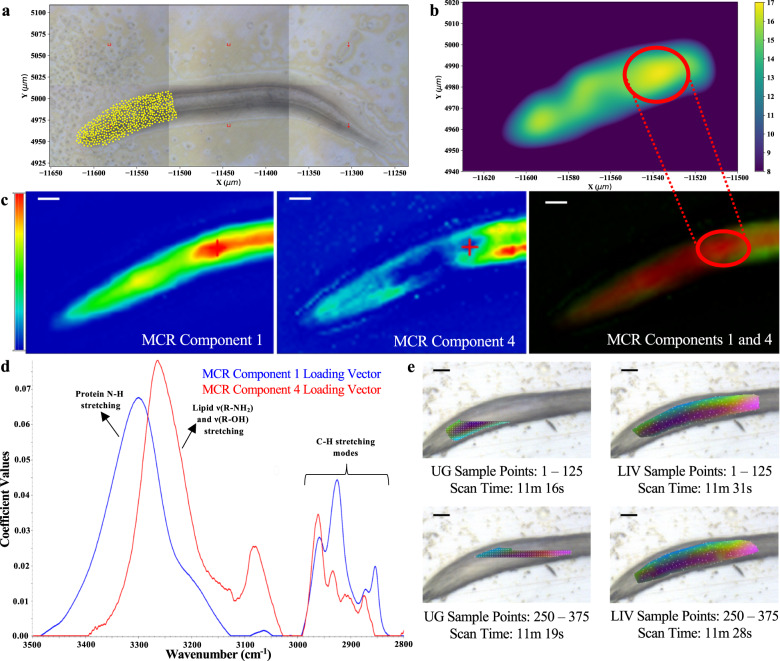
Application and performance evaluation of autonomous adaptive LIV data acquisition in the living nematode model system *Caenorhabditis elegans*. **a** Adaptive LIV data acquisition of an early-stage L2 *C. elegans* over a spatial map defined with domain knowledge to include the pharyngeal, nerve ring, and intestinal regions of the animal. **b** Density plot of sampled areas from adaptive LIV sampling for ease of more frequently sampled regions (red circle). **c** Coefficient heat maps of MCR component 1 (intensity range: 0.00–5.08), MCR component 4 (intensity range: 0.00–1.13), and the overlaid RG coefficient maps of both MCR components 1 and 4. Red cursor indicates same pixel; red circle indicates same region of dense sampling (scale bar, 10 μm). **d** Loading vectors for MCR components 1 (blue) and 4 (red) over evaluated frequency domain of 3500 to 2800 cm^−1^. Vibrational stretching assignments are labeled as discussed in the main text and Supplementary Fig. 7. **e** Standard UG sampling compared with LIV sampling over a tightly bound, pharyngeal mapping region of late-stage L1 *C. elegans* (scale bar, 10 μm) per given time interval as defined by ordered, sample point domain. The first three principal components are displayed as RGB false color composites.

For our proof-of-principle bioimaging case, we applied scanning broadband SR-FTIR spectromicroscopy to overcome signal-to-noise limitations when characterizing a young L2 *C. elegans* animal. *Caenorhabditis elegans* are well characterized in genetics, microscopy, and omics-related fields while also representing a large, whole-organism experimental model containing known compartmentalized chemistry. Relative to the diffraction-limited spatial resolution (2–10 μm) of scanning SR-FTIR spectromicroscopy, their large size of 100 μm to 1 mm in length when coupled with current mapping region software restrictions often lead to temporally inefficient spatiochemical mapping of unfixed samples. With our implemented user interface (Supplementary Fig.e 6), we were able to apply domain knowledge in spatial and spectral restrictions to better optimize our adaptive data acquisition of *C. elegans* (Fig. 2a) for comparison against the high-spatial (step-size 1.5 μm) resolution map of the same sample. We found that increased adaptive LIV sampling in the spatial domain (Fig. 2b) identified regions characterized by chemistries consistent with those of known anatomical features. Sampling increased in either transitional or overlapping anatomical regions between pharyngeal, head, neck, and body wall muscle^23^, regions of the nerve ring^24^, and the lipid-rich intestine^25^. Our qualitative post validation of adaptive data acquisition using multivariate curve resolution^26^ (MCR) and Fourier self-deconvolution^27^ (FSD) SR-FTIR analyses further confirmed these anatomical co-localization results with reliable MCR components^28^ 1 and 4 (Fig. 2c) corresponding to hydrated proteins (amino acid *ν*(N-H) stretching modes)^19^ and hydrated lipid assemblies (N-H, O-H, methyl, and *ν*(-(CH_2_)_*n*_-) stretching modes)^19,29^, respectively (Fig. 2d and Supplementary Fig. 7). With these two components overlapping in the more frequently sampled region, we verify that adaptive LIV data acquisition helps resolve spatiochemical gradients in a complex whole-organism model.

## Discussion

We constructed and implemented grid-less adaptive LIV data acquisition to address a key challenge in the hyperspectral imaging of time-sensitive systems. Specifically, we decrease image acquisition time while improving sampling density in regions of increased spatiochemical complexity. Using this sampling strategy, we non-destructively explore the chemistry of anatomical features in living *C. elegans*. We observe that increased sampling density corresponded with known anatomical features, and these results serve as a proof-of-principle for the use of AADA on a complex, biological specimen.

In this study, all experimental LIV-based AADA cases were performed on standard hardware found with commercial high-dimensional imaging microscope designs, revealing the accessibility and computational efficiency of the algorithm for a broadened use in imaging techniques that require a sequential exploration of space, such as scanning probe techniques. We show that LIV-based AADA can operate efficiently and effectively under conditions where the map area is unconstrained, and therefore, when the main goal of a study is to characterize a system through a discovery approach. This performance implies that LIV-based AADA will still benefit an experimentalist who has a detectable, discovery aspect of their research in an otherwise well-characterized biological systems that can range from single living cells to, in the case of smaller animal models like *C. elegans*, whole organisms. We also report that LIV-based adaptive sampling outperforms standard sampling methodologies in complex biological systems in which we apply domain knowledge to restrict mapping regions. Specifically when referencing instrument time usage to spatiochemically image the young L2 *C. elegans* experimental case, we were able to map the head region in 45 min with the LIV-based AADA software in comparison to ~4.9 h with the commercially available software. Lastly, we find that LIV-based AADA provides more comprehensive spatiochemical understanding of the total map domain at any given time interval in comparison to the established and standard UG sampling (Fig. 2e), suggesting that this aspect can be harnessed for further development of AADA to achieve adaptive high-dimensional real-time, non-invasive, label-free imaging through modular additions to the sampling algorithm.

This advance in hyperspectral imaging offers the biological community an orthogonal perspective into the dynamic physico-chemical architectures of studied tissues and model organisms. Critically, this information can potentially guide an investigator towards time-points and regions of interest for follow-up omics characterization, which is important in but not limited to the areas of carcinogenesis and developmental biology. Particularly in cases of discovery-based experimental design, AADA enables unbiased assessment of spatially resolved chemical changes between biological samples that differ by genotype, drug treatment or substance exposure, and physiological state such as age. More broadly, LIV-based AADA can be applied to fields outside of biology, such as hyperspectral remote sensing and space exploration. In these cases, future development towards real-time AADA will enable rapid identification, characterization, and even surveillance of chemical spills, toxic algal bloom formation, and spontaneous solar events.

## Methods

### Autonomous adaptive sampling

Our adaptive sampling workflow is based upon LOOCV and begins with an initial scan of randomly distributed points. Using PCA for dimensionality reduction, frequency domain restriction, and rubber-band baseline correction in our IR preprocessing module, we increased computational and temporal efficiency by calculating and operating over the first five principal components during our proof-of-principle, temporally intensive, high-dimensional data acquisition. A model *U*_0_ based upon barycentric linear interpolation (LIV) is constructed from this processed data set. We quantify the sensitivity of the surrogate model to the removal of an individual data point through the *ϵ*_LOO_. By removing a single point *X*_*i*_, model *U*_−*i*_ is rebuilt using the incomplete data set. The *ϵ*_LOO_ associated with the sample point is the difference between the two models evaluated at the removed point $\delta \left({U}_{0}({X}_{i}),{U}_{-i}({X}_{i})\right)$ with respect to the *L*_2_ norm^30^. After this is iterated for every sampled point in the acquired data set, the region defined by the sampled point with highest *ϵ*_LOO_ is sampled next by picking a random point within that region. This procedure is repeated until a set criterion is reached, which in our case was 500 total sampled points.

To assess algorithm sampling performance, we aggregate the *ϵ*_LOO_ of all sample points in the acquired data set and quantify the self-consistency using established LOOCV^31^. We take the mean *ϵ*_LOO_, ${\langle {\epsilon }_{\mathrm{LOO}}\rangle }_{V}\,$, of all sample points in the data set and use it as an unbiased, quantitative measure of the model accuracy due to theoretical guarantees of ${\langle {\epsilon }_{\mathrm{LOO}}\rangle }_{V}$ convergence to a model’s generalization error^32^. Since acquired points are often neither regularly nor uniformly distributed in the case of adaptive sampling, we partition the region into a collection of cells {*V*_*i*_} containing positions closest to each point {*X*_*i*_}. The mean is then weighted by the associated Voronoi area of sample point {*X*_*i*_}. Explicitly, we define

1 $${\langle {\epsilon }_{\mathrm{LOO}}\rangle }_{V}=\frac{{\sum }_{i}| {V}_{i}| \cdot {\epsilon }_{{\mathrm{LOO}},i}}{{\sum }_{i}| {V}_{i}| }.$$

With the LOOCV adaptive sampling procedure, we follow the heuristic for ${\langle {\epsilon }_{\mathrm{LOO}}\rangle }_{V}$ minimization, and thus, effectively achieve minimization of model generalization error by sampling near the point with the largest *ϵ*_LOO_.

### Surrogate modeling

We use the scipy.interpolate.griddata method from the Python Scipy package to implement 2D barycentric linear interpolation and treat each PCA component independently. Although it is computationally efficient, it does not quantify uncertainty in error estimate. To address this, we include the Voronoi area associated with each point into our calculated *ϵ*_LOO_ by treating it as an ad hoc regularizer. For a collection of points ${\bf{X}}=\{{{\bf{x}}}_{i}\in {{\mathbb{R}}}^{d}\}$, the Voronoi cell that we associate with point **x**_*i*_ is the region of space containing positions in Euclidean distance closest to **x**_*i*_:

2 $${V}_{i}=\{u\in {{\mathbb{R}}}^{d}:| | {{\bf{x}}}_{i}-{\bf{u}}| {| }_{2}<| | {{\bf{x}}}_{j}-{\bf{u}}| {| }_{2}\,\forall j\ne i\}\subset {{\mathbb{R}}}^{d}.$$

The Voronoi area is the area of the set, ${{\mathcal{V}}}_{i}=| | {V}_{i}| |$. This implies that if point **x**_*i*_ is spatially isolated from the rest of the data set, then point **x**_*i*_ will be associated with a greater Voronoi area. By approximating the error uncertainty using the Voronoi area, we make use of the fact that linear interpolation error tends to increase with larger distances from points used in the interpolation. To achieve this, we first normalize both *ϵ*_LOO_ and ${{\mathcal{V}}}_{i}$ in order to compare both quantities using a linear scaling from [0, 1]:

3 $$\sigma ({X}_{i})=\frac{{X}_{i}-\min ({\bf{X}})}{\max ({\bf{X}})-\min ({\bf{X}})}.$$

Next, we take the regularized LIV *ϵ*_LOO_ to be

4 $${\epsilon }_{{\mathrm{LOO}},i}^{(\mathrm{LIV})}=\sigma ({\epsilon }_{{\mathrm{LOO}},i})+\sigma ({{\mathcal{V}}}_{i}),$$

which is used to calculate our adjusted *ϵ*_LOO_ for our adaptive data acquisition in simulations and experiments^33^. This technique is inspired by and related to the LOLA-Voronoi and CV-Voronoi surrogate modeling techniques^11,14^.

### Simulations

One thousand simulations were conducted on each of the 11 SR-FTIR maps of *C. elegans* with raster-scanned step resolution ranging from 1 to 5 μm for a total of 11,000 simulations per simulated sampling method (analysis of these datasets beyond the benchmarking here will be described elsewhere; Elizabeth A. Holman et al., in preparation). Sampling was simulated by retrieving subsets of data from the full-resolution maps. Assessed sampling procedures were non-adaptive UG, UR, LUR, and adaptive LIV sampling.

UG sampling takes measurements over a static, pre-defined rectangular grid. For our subsampling procedure, every *k* points were selected to produce a lower resolution grid with roughly 1/*k*^2^ fewer points. Simulation results were collected over all *k*^2^ possible subgrids, a set emerging from the lower resolution grid changing with the location of the first subsampled point. In the case of UR, LUR, and LIV subsampling, we used spectral data from the spatially closest grid point in the high-resolution SR-FTIR map to reasonably approximate the spectral information at the determined subsampled point. This approximation holds true when the number of subsampled points is significantly smaller than the total number of points contained within the SR-FTIR map. UR subsampling drew measurements from *k* uniformly random positions. At each iteration for every *k* points, LUR subsampling collected data from the most sparsely sampled region within the defined map boundary. For LIV subsampling, we use the previously described procedure (see “Surrogate modeling” above) and iterate for every *k* point.

Each sampling procedure performance was quantitatively evaluated using the ground truth error (*ϵ*_GT_) of the interpolation to its corresponding high-resolution map. *ϵ*_GT_ was calculated by measuring the root mean-squared error (RMSE) of the data subset’s interpolation to the spatially resolved and complete SR-FTIR *C. elegans* map, which we treated as our “ground truth.” We use linear interpolation for models *U*_UG_, *U*_LUR_, and *U*_UR_, but we construct *U*_LIV_ from a linear interpolation of sampled points. Every model *U* is built from a collection ${\{{{\bf{X}}}_{i}\}}_{i = 1,\ldots ,{N}_{\mathrm{s}}}\in {{\mathbb{R}}}^{2}$ of *N*_s_ sample points with spectra ${\{{{\bf{Y}}}_{i}\}}_{i = 1,\ldots ,{N}_{\mathrm{s}}}\in {{\mathbb{R}}}^{{N}_{\mathrm{f}}}$, where *N*_f_ is the number of spectral dimensions. We denote the points in the “ground truth” map ${\{{{\bf{X}}}_{i}^{(0)}\}}_{i = 1,\ldots ,{N}_{0}}$, with spectra $\{{{\bf{Y}}}_{i}^{(0)}\}$, where *N*_0_ is the number of samples taken. Since we assume that high-spatial resolution SR-FTIR maps are our “ground truth,” we can assume *N*_0_ ≫ *N*_s_ and aim for *U* to be a good model in that

5 $$U({{\bf{X}}}_{i}^{(0)})\approx {{\bf{Y}}}_{i}^{(0)}.$$

With this assumption, we define *ϵ*_GT_ as a metric of merit to be the RMSE of the model when compared to the “ground truth”:

6 $$\langle {\epsilon }_{\mathrm{GT}}\rangle =\sqrt{\mathop{\sum }\limits_{i}^{{N}_{0}}| | U({{\bf{X}}}_{i}^{(0)})-{{\bf{Y}}}_{i}^{(0)}| {| }_{2}^{2}}.$$

### Sample preparation

All samples were mounted on 0.5-mm-thick ZnSe crystals, which were cleaned with Milli-Q water, 5% acetic acid, acetone, then Milli-Q water sequentially in order to remove organics while minimizing crystal damage. The two-component control sample was prepared with high vacuum grease (2021854-1993, Dow Corning) that was lightly applied to a 0.5-mm-thick ZnSe crystal (CAS# 1315-09-9, International Crystal Laboratories) in an area identifiable by fiducial markings drawn with a permanent marker (Item #37003, Sanford Ultra-Fine Blue Sharpie Permanent Marker). Spectral standards were acquired of both components independently prior to autonomous adaptive sample acquisition of abiotic two-component system. Spectral regions of component mixing could be identified by alcohol presence in the mixed spectra.

The first *C. elegans* (N2; Caenorhabditis Genetics Center) animal used for temporal exploratory LIV experiments was selected at the late L1 stage (based on morphology). The second animal for qualitative LIV assessment via FTIR spectral analysis was selected at the young L2 stage (based on morphology). Each animal was moved from their agar growth plates to 1 μL of 0.25 mM Levamisole (CAS# 16595-80-5, Sigma-Aldrich) on the ZnSe crystal and rinsed three times with Milli-Q water before mounting the sample onto the microscope stage for imaging.

### Instrumentation

Scanning benchtop and synchrotron FTIR measurements were performed on a Nicolet Nic-Plan IR microscope with a ×32, 0.65 numerical aperture objective with a Thermo Scientific Nicolet iS50 FTIR spectrometer using a KBr beamsplitter and MCT (HgCdTe) detector at Beamline 1.4.3 of the Advanced Light Source at Lawrence Berkeley National Laboratory. Adaptive sampling was implemented using a GUI (Supplementary Fig. 6) developed in PyQt and installed on the Beamline 1.4.3 computer (Dell Optiplex 7050: 8 GB RAM, Intel Core i5-7500 CPU @ 3.41 GHz, Windows 10 64 bit). OMNIC 9.8 software by Thermo Fisher Scientific controlled the microscope and FTIR bench, and our software communicated with OMNIC through Dynamic Data Exchange to store the OMNIC background-subtracted spectral output into our software’s dataframe format.

In this study, we used two different infrared sources: an internal globar source and a synchrotron source. Although an internal globar source is readily available in commercial FTIR microscopes, an accelerator-based synchrotron source offers at least 1000 times improvement in brightness (photon counts per unit time per unit area) over the globar source^34^ at the same spatial resolution. As a result, we used different total co-added scan and spatial resolutions for measurements performed on each instrument, which is detailed in the following sections.

### Globar FTIR spectromicroscopy and multivariate analysis

Benchtop scanning FTIR measurements using internal globar source were performed in transmission mode with an aperture-limited spatial resolution of 75 μm × 75 μm. IR spectra between 650 and 4000 cm^−1^ at 4 cm^−1^ spectral resolution were collected with 16 co-added scans at a interferometer mirror velocity of 1.83 cm/s. Rubber-band baseline correction and dimensionality reduction via PCA to five components was performed over the entire collected spectral region during adaptive LIV data acquisition. For each experimental assessment of sampling method, the total sampled points were limited to 500 to remain below the full-resolution map of 840.

#### On-target ratio

We define OTR to be the number of samples that meet the on-target criteria over the total number of sampled points. To determine the criteria by which a spectrum is considered on-target, we use our full-resolution data set and remove spectra close to the detection limitations of the instrument that violate the signal-to-noise filter criteria. Using this noise-removed subset of data, we calculate the mean spectra of the noise-removed subset. After identifying one major peak component per known component standard, we evaluated all acquired spectra per method for the presence of either aforementioned peak above the threshold that we determined as the noise-removed mean intensity at defined frequencies to define OTR as

7 $${\mathrm{OTR}}_{\mathrm{method}}=\frac{{N}_{a\vee b}}{{N}_{\mathrm{total}}},$$

where *N*_*a*∨*b*_ is the number of spectra that met the either the first mean peak criterion, second mean peak criterion, or both mean peak criteria, while *N*_total_ is the total number of spectra acquired using the referenced data acquisition method. Using this definition of spectral metric, we calculated ${\mathrm{OTR}}_{\mathrm{LIV}}=\frac{474}{500}$ (0.95) and ${\mathrm{OTR}}_{\mathrm{UG}}=\frac{95}{500}$ (0.19).

#### FTIR multivariate analysis

The control sample components (high vacuum grease and permanent marker) were evaluated individually as spectral standards. The data were baseline corrected and vector normalized using OMNIC 9.8, and the spectral mean was calculated over eight spectra per standard. Referencing the normalized mean and variance spectra, we use domain knowledge to perform PCA over the frequency domains of 3600 to 2800 cm^−1^ and 1750 to 1450 cm^−1^ simultaneously before applying LDA to maximize interclass variance over intraclass variance of our factors^22^ of our baseline-corrected and vector-normalized data in MATLAB R2017a. 2D score plots were generated in which the nearness between classes indicates similarity, whereas distance implies dissimilarity. The final mean spectrum of each cluster is shown for spectral validation of vibrational modes, resulting in segregation of classes.

### Synchrotron FTIR spectromicroscopy and multivariate analysis

Scanning SR-FTIR diffraction-limited (2–10 μm) spectra were collected in transmission mode between 650 and 4000 cm^−1^ at 4 cm^−1^ spectral resolution and recorded with eight co-added scans at an interferometer mirror velocity of 6.3 cm/s. We restricted the spectral domain adaptive LIV sampling workflow from 900 to 3700 cm^−1^ to avoid signal contamination from detectable synchrotron noise and to decrease sample morphology^17^ baseline effects, respectively, on subsequent dimensionality reduction and error calculation steps. Rubber-band baseline correction and dimensionality reduction to five components was performed over the restricted spectral region between 900 and 4000 cm^−1^ during adaptive LIV sampling. Using domain knowledge, we restricted our mapping region to the pharynx, nerve ring, and intestine^35^ of our young L2 *C. elegans* to reduce off-target sampling with respect to *C. elegans* for increased temporal efficiency in spatiochemical mapping.

#### SR-FTIR multivariate analysis

We restricted our analyzed MCR domain from 3500 to 2800 cm^−1^ for reduction of morphological effects on the spectral baseline and for higher diffraction-limited spatial resolution, since the goal of MCR analysis was to qualitatively assess adaptive LIV data acquisition performance. Based upon the cumulative explained variance calculated by OMNIC 9.8 on our experimental data, we performed MCR analysis in OMNIC using five components in which 99.82% of data variance is explained. Guided by well-characterized *C. elegans* anatomy and chemistry, we identified reliable MCR components^28^ that would strongly correlate with muscle and lipid assembly structures—components 1 and 4. For better accuracy in peak identification on our MCR components, we applied FSD to the C-H vibrational region. Since our analysis region was restricted, we could only broadly state the presence of protein-related stretching vibrations of *ν*(N-H) from amino acids between 3390 and 3260 cm^−1^^19^ and polyglycine asymmetric CH_2_ stretching modes at  ~2925 cm^−1^^19^ (Supplementary Fig. 7) in MCR component 1. Similarly for MCR component 4 and in referencing characterized hydrated lipid assemblies, we found broad peak contributions from N-H and O-H stretching modes between 3400 to 3100 cm^−1^^29^, lipid-relevant antisymmetric *ν*(-(CH_2_)_*n*_-) modes at  ~2932 cm^−1^^29^, and lipid-related methyl antisymmetric and symmetric stretching at 2963 and 2873 cm^−1^^29^, respectively.

### Statistics and reproducibility

Each sample size, type, and statistical method applied is described in the relevant “Method” section. For the two-component model system, spectral standards for permanent ink and high vacuum grease were performed with sample replicates (*n* = 8).

### Reporting summary

Further information on research design is available in the Nature Research Reporting Summary linked to this article.

## Supplementary information

Supplementary Information

Reporting Summary

## Data Availability

Infrared spectral data are available through the CaltechDATA repository (10.22002/D1.1609)^,36^. The 11 high-resolution spectral maps used for calibration simulations are not included in the repository, since they are undergoing spectral analysis and interpretation in a different manuscript. Any remaining data is available from the corresponding author upon reasonable request.

## Code availability

This proprietary adaptive sampling code and GUI are specific to the Infrared Beamline 1.4.3 at the Advanced Light Source (https://als.lbl.gov/). They are available to IR beamline users through the DOE-supported Berkeley Synchrotron Infrared Structural Biology (BSISB) Imaging Program (https://bsisb.lbl.gov/wordpress/). Further requests concerning this code can be directed to H.-Y.N.H.

## References

[1] Liu T (2018). Observing the cell in its native state: imaging subcellular dynamics in multicellular organisms. Science.

[2] Chalfie M, Tu Y, Euskirchen G, Ward WW, Prasher DC (1994). Green fluorescent protein as a marker for gene expression. Science.

[3] Giepmans BNG, Adams SR, Ellisman MH, Tsien RY (2006). The fluorescent toolbox for assessing protein location and function. Science.

[4] Bernd B (2016). Multiplexed epitope-based tissue imaging for discovery and healthcare application. Cell Syst..

[5] Choi M, Kwok SJJ, Yun SH (2015). In vivo fluorescence microscopy: lessons from observing cell behavior in their native environment. Physiology.

[6] Prahst C (2020). Mouse retinal cell behaviour in space and time using light sheet fluorescence microscopy. eLife.

[7] Zhao M (2006). Electrical signals control wound healing through phosphatidylinositol-3-OH kinase-gamma and PTEN. Nature.

[8] Rohban MH, Abbasi HS, Singh S, Carpenter AE (2019). Capturing single-cell heterogeneity via data fusion improves image-based profiling. Nat. Commun..

[9] Asprey SP, Macchietto S (2002). Designing robust optimal dynamic experiments. J. Process Control.

[10] Queipo N (2005). Surrogate-based analysis and optimization. Prog. Aerosp. Sci..

[11] Crombecq, K., De Tommasi, L. D., Gorissen, D. & Dhaene, T. A novel sequential design strategy for global surrogate modeling. In *Proc. 2009 Winter Simulation Conference (WSC)*, 731–742 (2009).

[12] Li G, Aute V, Azarm S (2010). An accumulative error based adaptive design of experiments for offline metamodeling. Struct. Multidiscip. Optim..

[13] Wang C (2014). An evaluation of adaptive surrogate modeling based optimization with two benchmark problems. Environ. Model. Softw..

[14] Xu S, Liu H, Wang X, Jiang X (2014). A robust error-pursuing sequential sampling approach for global metamodeling based on voronoi diagram and cross validation. J. Mech. Des..

[15] Singh, P., Deschrijver, D. & Dhaene, T. A balanced sequential design strategy for global surrogate modeling. In *Simulation Conference (WSC), 2013 Winter*, 2172–2179 (IEEE, 2013).

[16] Elisseeff A, Evgeniou T, Pontil M (2006). Stability of randomized learning algorithms. J. Mach. Learn. Res..

[17] Baker MJ (2014). Using Fourier transform IR spectroscopy to analyze biological materials. Nat. Protoc..

[18] Lippincott ER, Van Valkenburg A, Weir CE, Bunting EN (1958). Infrared studies on polymorphs of silicon dioxide and germanium dioxide. J. Res. Natl Bur. Stand..

[19] Socrates, G. *Infrared and Raman Characteristic Group Frequencies* (Wiley, 2001).

[20] Awab H, Jar ADM, Yong WK, Ahmad UK (2011). Infrared spectroscopic technique for the forensic discrimination of marker pen inks. Malays. J. Forensic Sci..

[21] Razavi S, Tolson BA, Burn DH (2012). Review of surrogate modeling in water resources. Water Resour. Res..

[22] Hu P (2013). Metabolic phenotyping of the cyanobacterium Synechocystis 6803 engineered for production of alkanes and free fatty acids. Appl. Energy.

[23] Altun, Z. F. & Hall, D. H. in *WormAtlas*10.3908/wormatlas.1.6 (2009).

[24] Altun, Z. F. & Hall, D. H. in *WormAtlas*10.3908/wormatlas.1.1 (2009).

[25] Mak HY (2012). Lipid droplets as fat storage organelles in *Caenorhabditis elegans*. J. Lipid Res..

[26] Felten J (2015). Vibrational spectroscopic image analysis of biological material using multivariate curve resolution? Alternating least squares (MCR-ALS). Nat. Protoc..

[27] Tooke PB (1988). Fourier self-deconvolution in IR spectroscopy. Trends Anal. Chem..

[28] Motegi H (2015). Identification of reliable components in multivariate curve resolution-alternating least squares (MCR-ALS): a data-driven approach across metabolic processes. Sci. Rep..

[29] Mantsch, H. H. & Chapman, D. (eds). *Infrared Spectroscopy of Biomolecules* (Wiley-Liss, 1995).

[30] Ramirez-Lopez L (2013). Distance and similarity-search metrics for use with soil vis?NIR spectra. Geoderma.

[31] Cawley GC, Talbot NLC (2003). Efficient leave-one-out cross-validation of kernel fisher discriminant classifiers. Pattern Recogn..

[32] Kearns M, Ron D (1999). Algorithmic stability and sanity-check bounds for leave-one-out cross-validation. Neural Comput..

[33] Bandler JW (2004). Space mapping: the state of the art. IEEE Trans. Microw. Theory Tech..

[34] Holman HN, Martin MC, McKinney WR (2003). Tracking chemical changes in a live cell: biomedical applications of SR-FTIR spectromicroscopy. Spectroscopy.

[35] Altun, Z. F. & Hall, D. H. in *WormAtlas*10.3908/wormatlas.1.1 (2009).

[36] Holman, E. Dataset for Autonomous Adaptive Data Acquisition (AADA) (Version 1.0). *CaltechDATA.*10.22002/D1.1609 (2020).

