# Supplementary material for: Autonomous adaptive data acquisition for scanning hyperspectral imaging
Source: Commun Biol. 2020 Nov 18;3:684. doi: 10.1038/s42003-020-01385-3 (PMC7676237; doi:10.1038/s42003-020-01385-3)
Supplement: Supplementary file 1 — Supplementary Information [file 42003_2020_1385_MOESM1_ESM.pdf]

Supplementary Information for

Autonomous Adaptive Data Acquisition for Scanning Hyperspectral Imaging

Elizabeth A. Holman†[1], Yuan-Sheng Fang†[2,3], Liang Chen[3], Michael DeWeese[2], Hoi-Ying N. Holman[3], Paul W. Sternberg*[4]

[1]Division of Chemistry and Chemical Engineering, California Institute of Technology
[2]Department of Physics, University of California, Berkeley
[3]Division of Molecular Biophysics and Integrated Bioimaging, Lawrence Berkeley National Laboratory
[4]Division of Biology and Biological Engineering, California Institute of Technology

Supplemental Figures

Voronoi-weighted leave-one-out error mean for two-component sample 2

Globar FTIR mean spectra of model compounds for two-component sample 3

Globar FTIR variance spectra of model compounds for two-component sample 3

Globar FTIR spectral comparison of known permanent ink ingredients 4

Identified cluster mean spectra via exploratory PC-LDA 5

Implemented graphical user interface for autonomous adaptive data acquisition 6

Fourier self-deconvolution of target SR-FTIR MCR component loading vectors 7

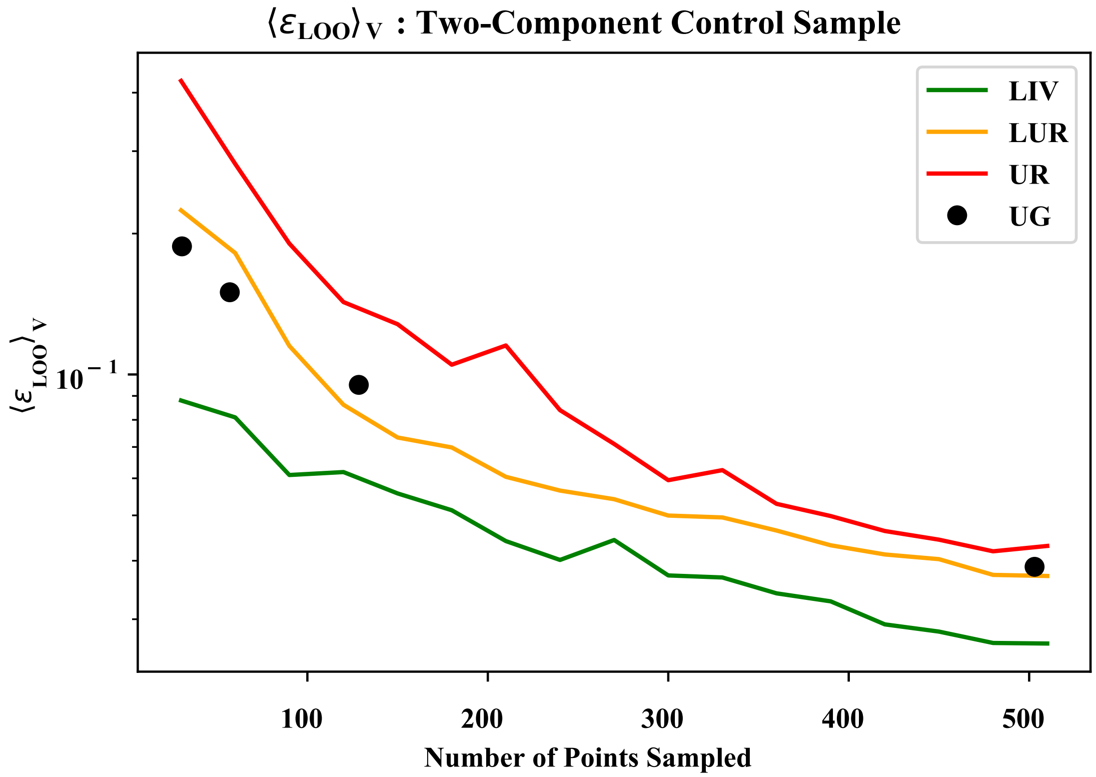

Supplemental Figure 1. Voronoi-weighted leave-one-out error mean plotted for each non-adaptive and adaptive data acquisition experiment for the abiotic two-component sample. Adaptive LIV data acquisition of map regions defined without domain knowledge outperforms its non-adaptive data acquisition counterparts.

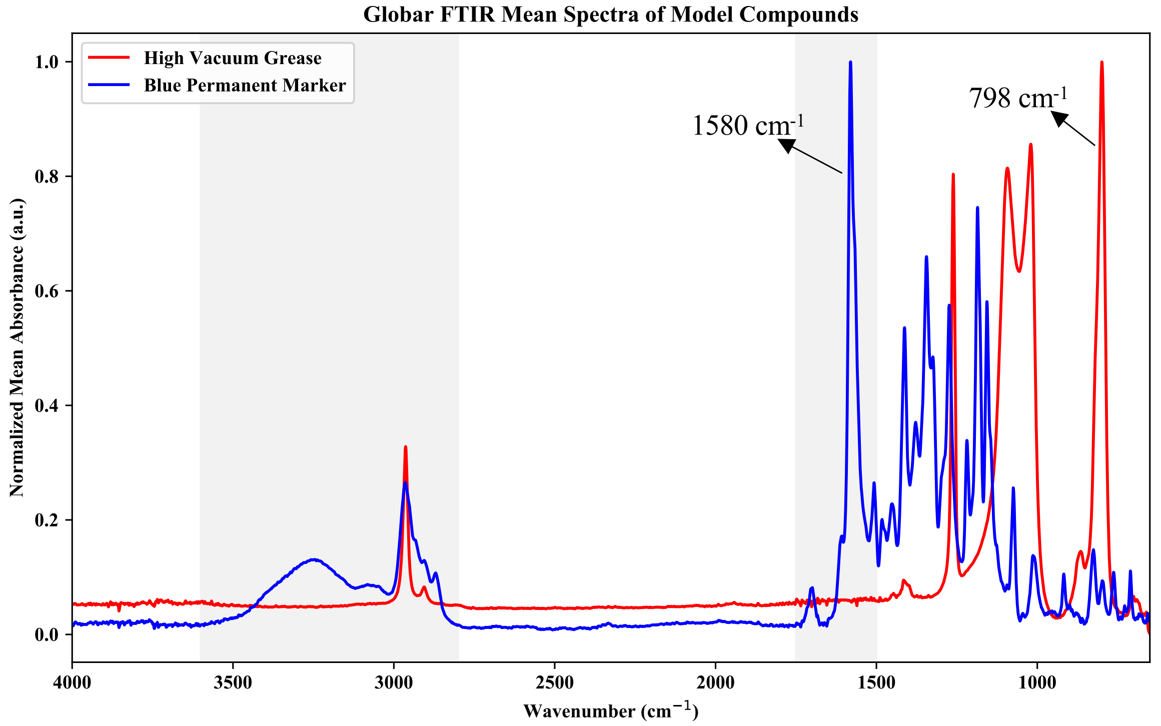

Supplemental Figure 2. Globar FTIR mean absorbance identity fingerprint spectra (N = 8) of each model compound eventually used in synthetic two-component control sample. On-target ratios were calculated using spectral peaks at 1580 cm^{-1} and 798 cm^{-1}, which were the major identification peaks of permanent marker and high vacuum grease respectively. PC-LDA regions (gray) were determined from mean and variance spectra.

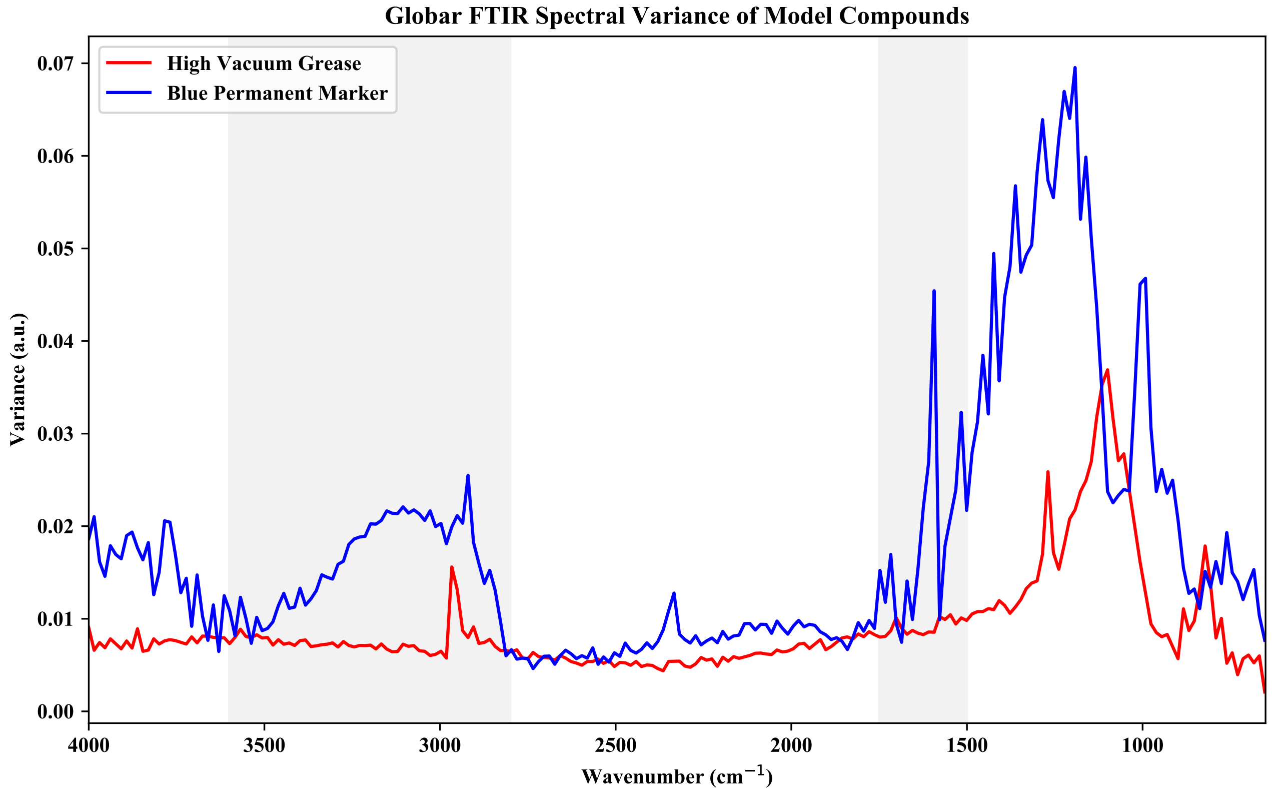

Supplemental Figure 3. Globar FTIR variance fingerprint spectra of each model compound eventually used in synthetic two-component control sample. The variance was used to determine the domains selected for exploratory PC-LDA (gray) when coupled with mean IR spectral information, as described in Online Methods.

Globar FTIR Spectral Comparison of Permanent Ink Ingredients

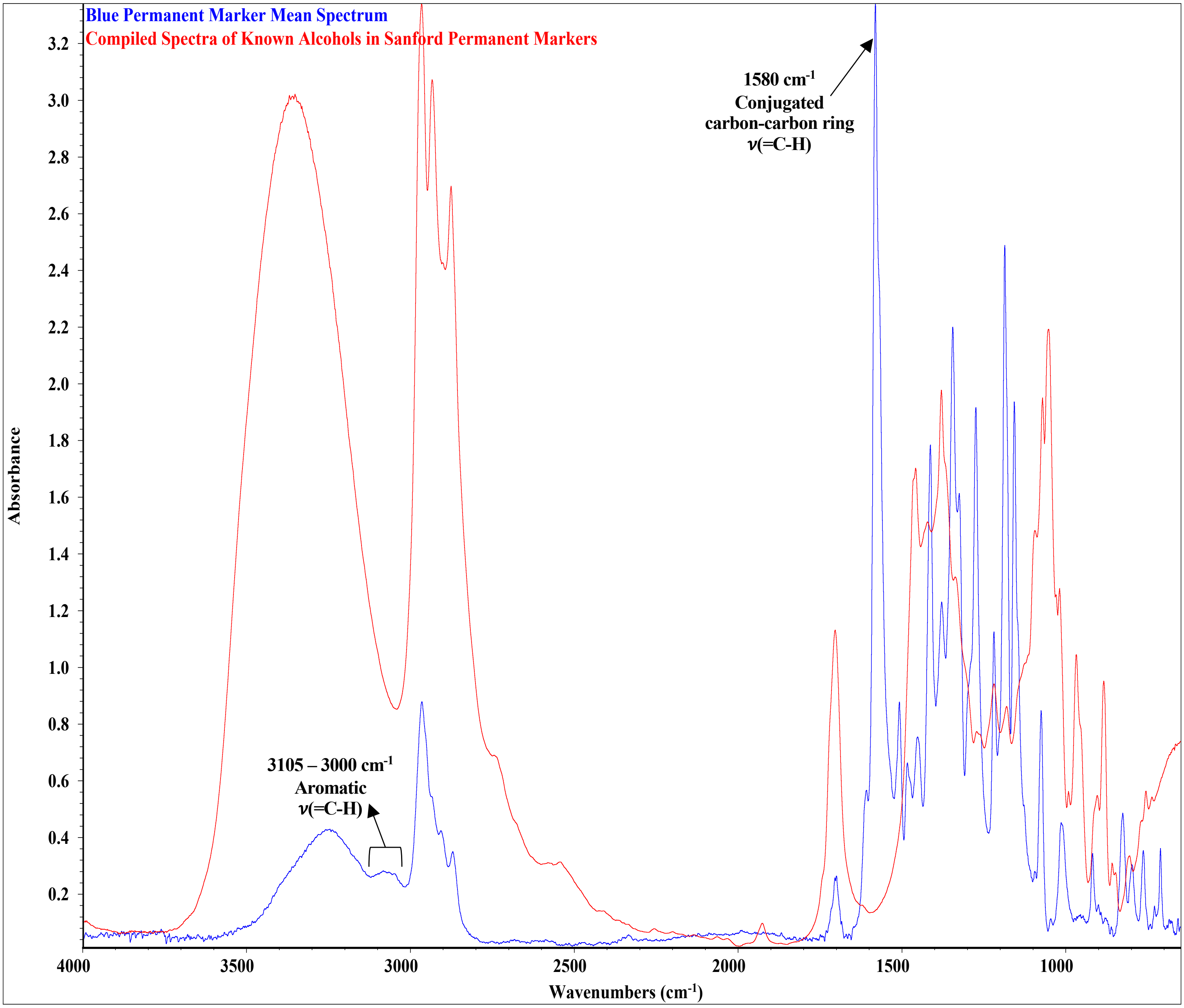

Supplemental Figure 4. Known alcohol ingredients of Sanford Permanent Marker ink shown as cumulative IR spectrum generated using OMNIC 9.8 alcohol libraries: ethyl alcohol, 1-propanol, 1-butanol, and diacetone alcohol in red to contrast against our normalized permanent marker ink mean spectrum obtained using globar FTIR spectromicroscopy. Identified peaks support the discussion and analysis of the main text presenting 1580 cm^{-1} and region 3105 cm^{-1} to 3000 cm^{-1} vibrational modes as unique to the pigments or dyes present in the deposited ink. Plotted in OMNIC 9.8.

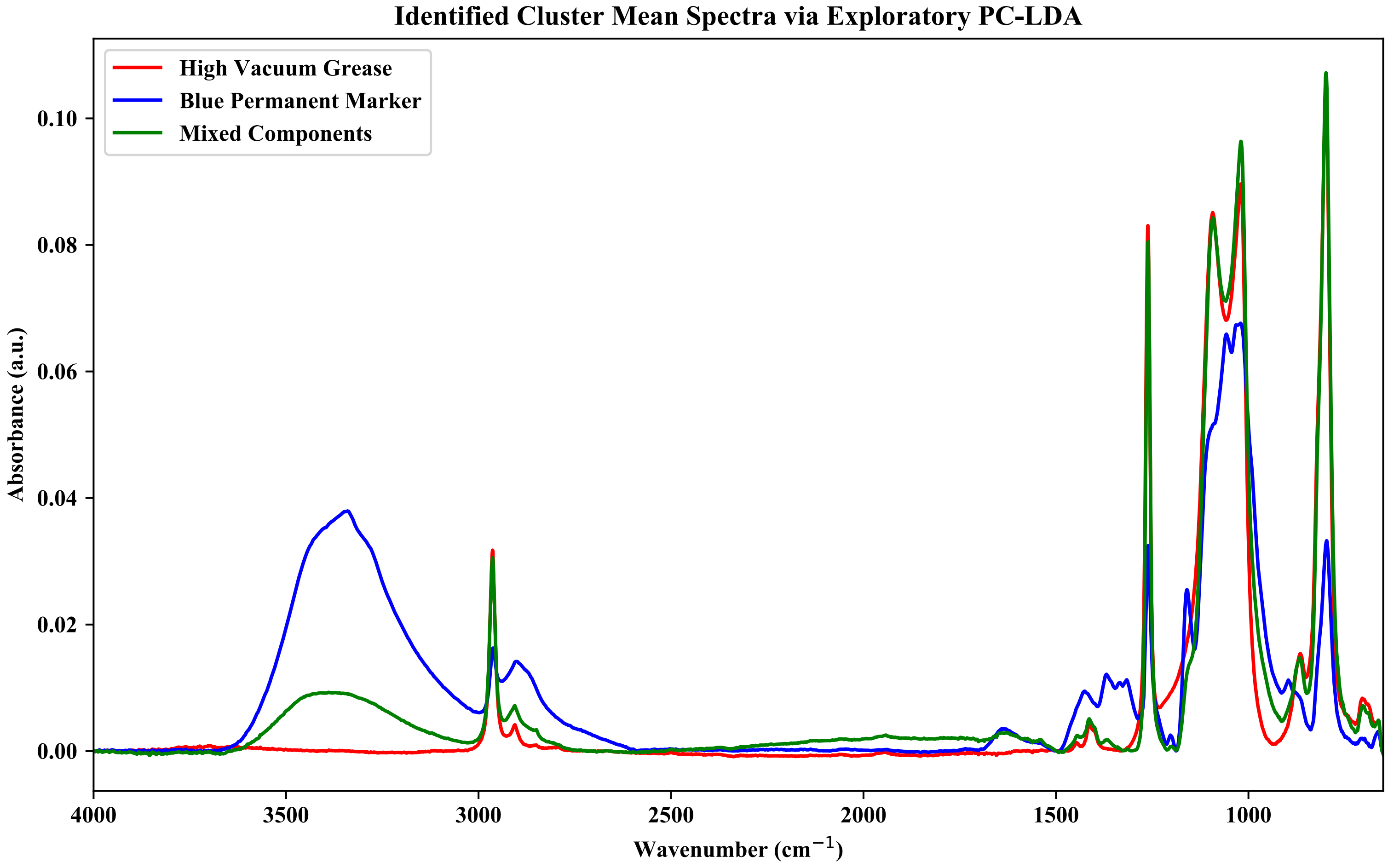

Supplemental Figure 5. Full spectral region for identified cluster mean spectra of baseline-corrected cluster spectra. Spectra were not normalized in order to conserve concentration information.

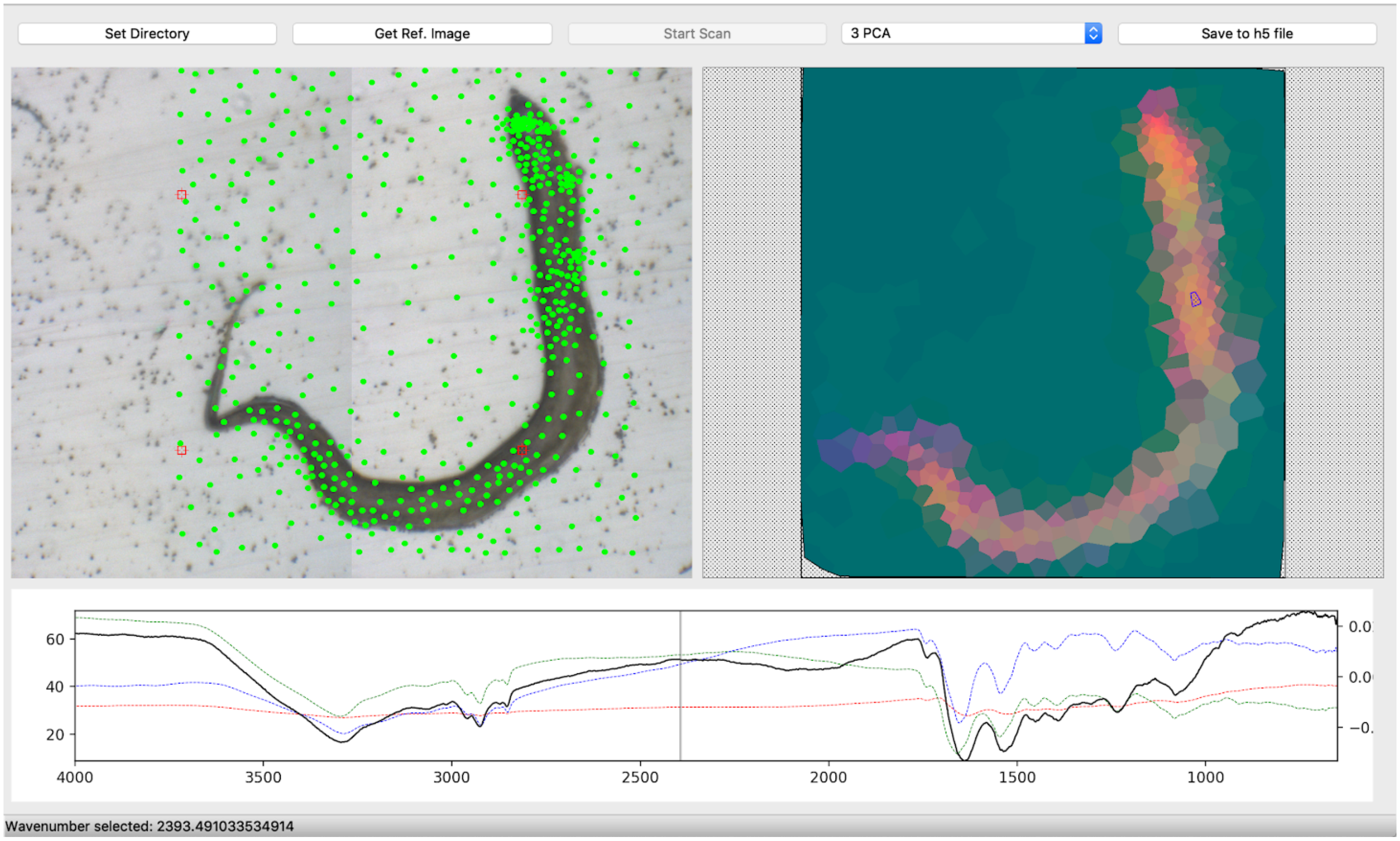

Supplemental Figure 6. Implemented graphical user interface for autonomous adaptive data acquisition at the Advanced Light Source's Beamline 1.4.3. Spectral output displayed is in transmission mode. Domain knowledge was not applied in the shown experimental *C. elegans* case. In left window, sampled points performed by adaptive data acquisition in real-time are shown as green circles; in right window, either PCA components 1-3 coefficients or a specific wavenumber is displayed as a heat map as data is acquired.

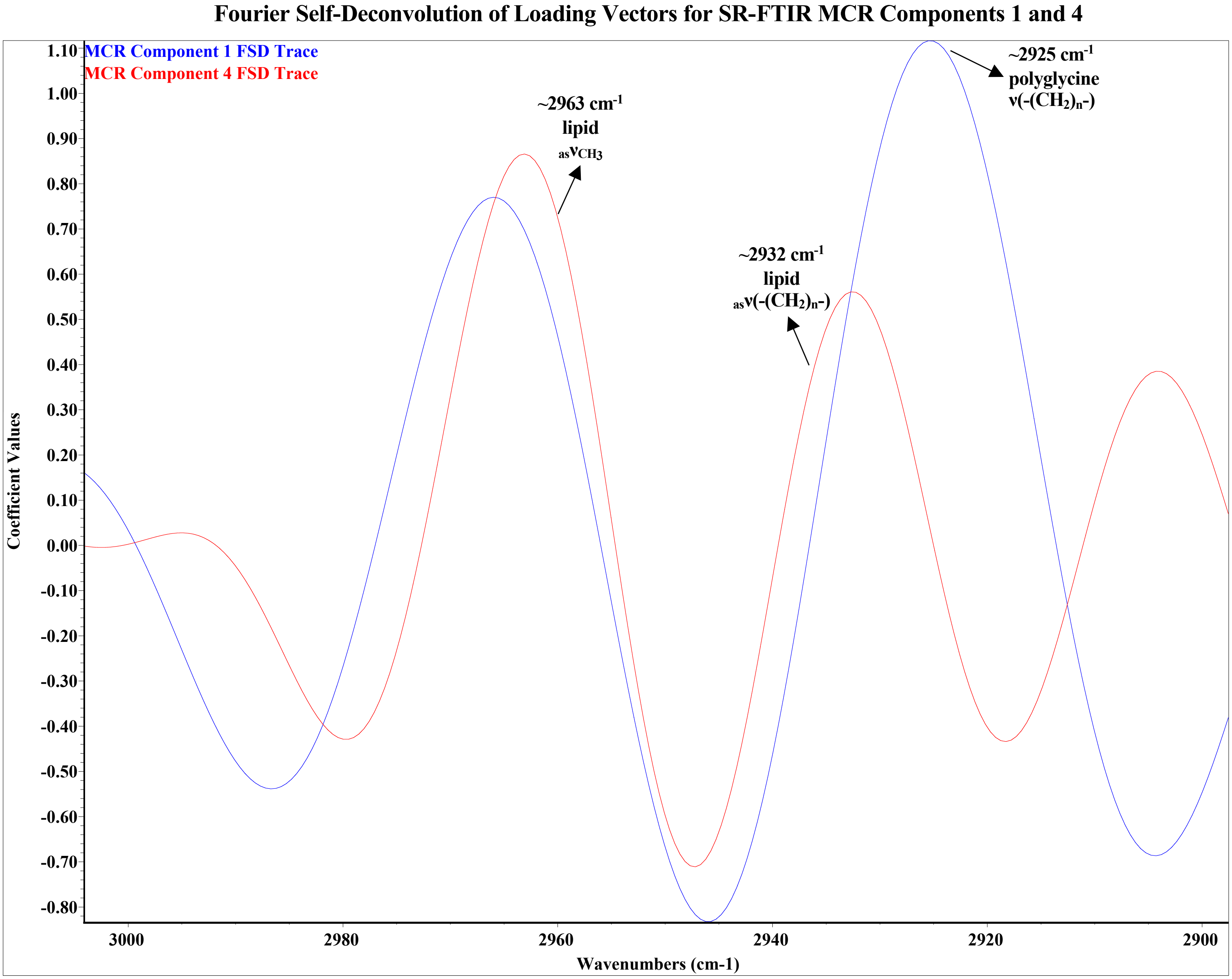

Supplemental Figure 7. FSD plots of the 3000 cm^{-1} to 2900 cm^{-1} spectral region for MCR components 1 and 4 for accurate peak identification in a high signal-to-noise region of C-H stretching vibrations. To be conservative, we only used major peaks to support assignments and co-localization with chemistry of known anatomical structures. For component 4, we referenced the asymmetric stretching mode of characterized lipid methyl groups (~2963 cm^{-1}) and lipid antisymmetric stretching $-(CH_2)_n-$ modes (2916 cm^{-1} – 2936 cm^{-1}). For component 1, we referenced the asymmetric stretching $-(CH_2)_n-$ mode of characterized biological polyglycines (~2925 cm^{-1}). Plotted in OMNIC 9.8.
