# Supplementary material for: Autonomous adaptive data acquisition for scanning hyperspectral imaging
Source: Commun Biol. 2020 Nov 18;3:684. doi: 10.1038/s42003-020-01385-3 (PMC7676237; doi:10.1038/s42003-020-01385-3)
Supplement: Supplementary file 2 — Reporting Summary [file 42003_2020_1385_MOESM2_ESM.pdf]

nature research

Corresponding author(s): Paul W. Sternberg

Last updated by author(s): Aug 22, 2020

Reporting Summary

Nature Research wishes to improve the reproducibility of the work that we publish. This form provides structure for consistency and transparency in reporting. For further information on Nature Research policies, see Authors & Referees and the Editorial Policy Checklist .

Statistics

For all statistical analyses, confirm that the following items are present in the figure legend, table legend, main text, or Methods section.

n/a	Confirmed	
☒	☐	The exact sample size (*n*) for each experimental group/condition, given as a discrete number and unit of measurement
☐	☒	A statement on whether measurements were taken from distinct samples or whether the same sample was measured repeatedly
☒	☐	The statistical test(s) used AND whether they are one- or two-sided<br>*Only common tests should be described solely by name; describe more complex techniques in the Methods section.*
☒	☐	A description of all covariates tested
☐	☒	A description of any assumptions or corrections, such as tests of normality and adjustment for multiple comparisons
☐	☒	A full description of the statistical parameters including central tendency (e.g. means) or other basic estimates (e.g. regression coefficient) AND variation (e.g. standard deviation) or associated estimates of uncertainty (e.g. confidence intervals)
☐	☒	For null hypothesis testing, the test statistic (e.g. *F*, *t*, *r*) with confidence intervals, effect sizes, degrees of freedom and *P* value noted<br>*Give P values as exact values whenever suitable.*
☒	☐	For Bayesian analysis, information on the choice of priors and Markov chain Monte Carlo settings
☐	☒	For hierarchical and complex designs, identification of the appropriate level for tests and full reporting of outcomes
☒	☐	Estimates of effect sizes (e.g. Cohen's *d*, Pearson's *r*), indicating how they were calculated

Our web collection on statistics for biologists contains articles on many of the points above.

Software and code

Policy information about availability of computer code

Data collection	Adaptive sampling was implemented using a GUI developed in PyQt and installed on the Beamline 1.4.3 computer. OMNIC 9.8 software by ThermoFisher Scientific controlled the microscope and FTIR bench, and our software communicated with OMNIC through Dynamic Data Exchange (DDE) to store the OMNIC background-subtracted spectral output into our software's dataframe format.
Data analysis	Data analysis for globar and synchrotron infrared spectromicroscopy was performed using MATLAB R2017a, python scripts for bulk data handling, and OMNIC 9.8.

For manuscripts utilizing custom algorithms or software that are central to the research but not yet described in published literature, software must be made available to editors/reviewers. We strongly encourage code deposition in a community repository (e.g. GitHub). See the Nature Research guidelines for submitting code & software for further information.

Data

Policy information about availability of data

All manuscripts must include a data availability statement. This statement should provide the following information, where applicable:

- Accession codes, unique identifiers, or web links for publicly available datasets
- A list of figures that have associated raw data
- A description of any restrictions on data availability

Infrared spectral data are available through the CaltechDATA repository (DOI: 10.22002/D1.1609). The eleven high-resolution spectral maps used for calibration simulations are not included in the repository, since they are being spectrally analyzed and interpreted in a different manuscript. The proprietary adaptive sampling code and GUI are specific to the Infrared Beamline 1.4.3 at the Advanced Light Source (https://als.lbl.gov/). They are available to IR beamline users through the DOE-supported Berkeley Synchrotron Infrared Structural Biology (BSISB) Imaging Program (https://bsisb.lbl.gov/wordpress/).

Field-specific reporting

Please select the one below that is the best fit for your research. If you are not sure, read the appropriate sections before making your selection.

- [x] Life sciences
- [ ] Behavioural & social sciences
- [ ] Ecological, evolutionary & environmental sciences

For a reference copy of the document with all sections, see nature.com/documents/nr-reporting-summary-flat.pdf

Life sciences study design

All studies must disclose on these points even when the disclosure is negative.

Sample size	n/a; 2 experimental cases; mean standard spectra for identification (n=8)
Data exclusions	Noise filter applied for infrared spectra obtained with globar FTIR spectromicroscopy.
Replication	Simulations were repeated as described in methods section. Mean spectra were obtained as described in methods section.
Randomization	Random spatial seeding for adaptive data acquisition was performed with 20 sample points.
Blinding	Essentially blinded sequential sampling due to automated adaptive nature of method.

Reporting for specific materials, systems and methods

We require information from authors about some types of materials, experimental systems and methods used in many studies. Here, indicate whether each material, system or method listed is relevant to your study. If you are not sure if a list item applies to your research, read the appropriate section before selecting a response.

Materials & experimental systems

n/a	Involved in the study	
x		Antibodies
x		Eukaryotic cell lines
x		Palaeontology
	x	Animals and other organisms
x		Human research participants
x		Clinical data

Methods

n/a	Involved in the study	
x		ChIP-seq
x		Flow cytometry
x		MRI-based neuroimaging

Animals and other organisms

Policy information about studies involving animals; ARRIVE guidelines recommended for reporting animal research

Laboratory animals	C. elegans
Wild animals	None
Field-collected samples	None
Ethics oversight	N/A

Note that full information on the approval of the study protocol must also be provided in the manuscript.
